# Bone Mineral Density (BMD) Assessment Using Dual-Energy CT with Different Base Material Pairs (BMPs)

**DOI:** 10.3390/jimaging11070236

**Published:** 2025-07-13

**Authors:** Stefano Piscone, Sara Saccone, Paola Milillo, Giorgia Schiraldi, Roberta Vinci, Luca Macarini, Luca Pio Stoppino

**Affiliations:** 1 Department of Medical and Surgical Sciences, Section of Diagnostic Imaging, University of Foggia, Viale Luigi Pinto n. 1, 71121 Foggia, Italy; sara.saccone@unifg.it (S.S.); paola.milillo@yahoo.it (P.M.); roberta.vinci@unifg.it (R.V.); luca.macarini@unifg.it (L.M.); luca.stoppino@unifg.it (L.P.S.); 2 Unit of Diagnostic and Interventional Radiology, Department of Radiology, Maggiore Polyclinic Hospital, Foundation IRCCS Ca’ Granda, 20122 Milan, Italy; giorgiaschiraldi1997@gmail.com

**Keywords:** bone mineral density (BMD), Dual-Energy CT (DECT), Dual-energy X-ray Absorptiometry (DXA), osteoporosis, femoral neck fractures, base materials pairs (BMPs)

## Abstract

The assessment of bone mineral density (BMD) is essential for osteoporosis diagnosis. Dual-energy X-ray Absorptiometry (DXA) is the current gold standard, but it has limitations in evaluating trabecular bone and is susceptible to different artifacts. In this study we evaluate whether Dual-Energy Computed Tomography (DECT) can be defined as an alternative method for the assessment of BMD in a sample of postmenopausal patients undergoing oncological follow-up. In this study a retrospective analysis was conducted on 41 patients who had both DECT and DXA within six months. BMD values were extracted from DECT using five different base material pairs (BMPs) and compared with DXA measurements at the femoral neck. The calcium–fat pairing showed the strongest correlation with DXA-derived BMD (Spearman’s ρ = 0.797) and excellent reproducibility (ICC = 0.983). There was a strong and significant association between the DXA results and the various BPM measurements. These findings support the possibility of DECT in the precise and opportunistic evaluation of BMD changes when employing particular BMPs. This study showed how this technique can be a useful and effective substitute for conventional DXA, particularly when patients are in oncological follow-up using DECT, minimizing additional radiation exposure.

## 1. Introduction

Alterations in bone mineral density (BMD) are a current and constantly growing issue, as they are related to the aging of the population. According to the WHO definition of osteoporosis, the disease affects approximately 6% of men and 21% of women over the age of 50 worldwide; that equates to an estimated 500 million people affected by osteoporosis [1,2].

With the increasing number of elderly populations worldwide, the incidence of osteoporosis and osteoporotic fractures has seen a steady increase [3]. In this population, proximal femoral fractures are among the most common fractures and have been well described in terms of clinical problems and epidemiological literature [4]. Osteoporotic femoral neck fracture is characterized by drastic morbidity, disability, and a reduction in quality of life. Furthermore, representing approximately 20% of all osteoporotic fractures, they are among the most destructive and associated with high healthcare costs [5]. In particular, in cases of osteoporotic femoral neck fractures, mortality rates in the 5 years following the event vary from 38 to 64% depending on age [6].

### 1.1. State of the Art

According to WHO, dual-energy X-ray absorbimetry (DXA) is the recommended method for diagnosing osteoporosis [7]. DXA uses a combination of two X-ray beams with different energy levels to assess an individual’s BMD. In particular, it uses the T-score to stratify risk classes into osteoporosis, osteopenia, and normal. This statistical value expresses how much the BMD of an individual deviates, in terms of standard deviations, from the average of a reference population consisting of healthy members of the same sex aged between 20 and 30 years. According to the criteria established by the World Health Organization (WHO), a value of T-score equal to or less than −2.5 indicates a condition of osteoporosis, while values between −1 and −2.5 are indicative of osteopenia. Studies have noted a discrepancy in T-score densitometry between the hip and lumbar spine, implying that the WHO cutoffs for osteoporosis and osteopenia may be inaccurate [8]. Furthermore, BMD values obtained with DEXA in healthy individuals have a coefficient of variation of 1–2% at the lumbar spine and 2–3% at the femoral neck. Therefore, precision bias must be considered when evaluating repeat DEXA scans to distinguish between true changes in bone density and possible random variations caused by variability in the measurement procedure [9].

However, it has limitations, including accurate detection of early changes in the spongy bone and its susceptibility to various artifacts (ligaments and vascular calcifications, degenerative bone changes, etc.) leading to false negative outcomes [10].

Recently, Dual-Energy Computed Tomography (DECT) has become widely used in routine clinical practice due to its ability to characterize tissue composition based on material-specific attenuation across different energy spectra.

It has various applications in musculoskeletal imaging, including the assessment of bone marrow edema in trauma, the diagnosis of vertebral fractures, and the identification of bone marrow tumors [11]. Material decomposition is based on the principle that attenuation images that are obtained at different X-ray voltage levels can be translated into density maps for two reference materials. In these maps, each voxel represents the density of the specific material at that image position [12].

Patino et al. have shown that absorption in different types of tissue depends on the percentage composition of base materials, suggesting that the selection of appropriate base material pairs (BMPs) allows for an accurate description of the material content of the tissue itself [13]. The choice of BMP requires further exploration due to the complex composition of the bone. In view of the above, the use of pairs of basic materials widely present in bone structures (such as hydroxyapatite, calcium, water and fat) can present different values based on their percentage presence in the examined region.

DECT is widely used for the follow-up of oncology patients, as its virtual non-contrast imaging helps reduce radiation exposure [14,15], so the integration of BMD assessment during oncological follow-up with DECT could further reduce the radiation dose by minimizing the need for separate DXA scans. Phantom studies have shown that density measurements of DECT materials are linearly related to real concentrations in two-material models [16]. In addition, a strong linear correlation was found between the bone mineral density (BMD) values obtained by DECT and those obtained with the DXA technique. This suggests that DECT could be a good opportunity for the quantification of BMD on scans routinely performed for other clinical purposes [17].

### 1.2. Aim of the Study

To date, few studies have been conducted comparing different BMPs in order to evaluate which of these had a better correlation with the gold standard (DXA).

In view of the above, we performed in our study a base material pairing of the major components of the bone and analyzed five base material pairs: carboxymonohydrated–water, calcium–fat, calcium–water, hydroxyapatite–fat, and hydroxyapatite–water.

The aim of this study is to evaluate the potential of DECT for the assessment of BMD at the femoral neck, analyzing data obtained from different pairs of materials (BPMs) and the corresponding BMD values obtained from the gold standard (DXA), in order to identify which pair presents the highest concordance with BMD measurements obtained by DXA.

## 2. Materials and Methods

### 2.1. Study Population

This retrospective opportunistic study included 41 postmenopausal women who had both BMD assessment and an oncologic follow-up with DECT. Only patients with a maximum interval of six months between DXA and DECT were included in the study, excluding cases with bone lesions or metal prostheses (Table 1).

### 2.2. Imaging

DECT images were acquired using a 256-slice spectral multidetector CT system (Revolution, GE Healthcare, Chicago, IL, USA). The scans were performed with a 1.0 mm slice thickness and a tube current of 200 mA with the Dose Right automatic exposure control system. Conventional images at 80 and 140 kVp were initially reconstructed at a 0.625 mm thickness using a soft-tissue kernel, followed by further post-processing.

All data obtained by CT images were reconstructed using the Gemstone Spectral Imaging (GSI) technique, with multiplanar reconstructions (MPRs) generated in coronal and sagittal planes. Virtual monochromatic images were derived from the portal venous phase, obtained through rapid kVp-switching DECT (80/140 kVp) with GSI set at a pitch of 0.984 and a rotation time of 0.8 s. Contrast enhancement was achieved via a weight-adjusted injection (1.4 mL/kg) of iohexol 350 mgI/mL, administered at a rate of 3.5 mL/s. To minimize vascular artifacts, regions adjacent to blood vessels were excluded from measurements, and bone segments affected by any types of artifacts were not included in the analysis.

**Figure 1:**
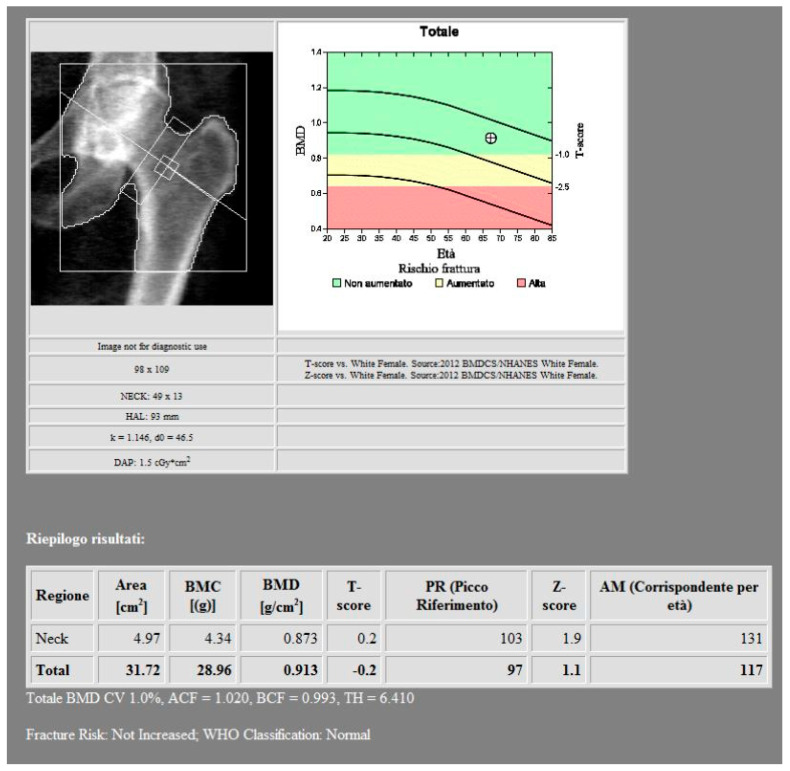
DXA measurements on the femoral neck (Regione: measurement region; Area: measurement area; BMC: bone mineral content; BMD: bone mineral density; PR: reference peak; AM: age matched).

BMD measurements were made using DXA with a Discovery A bone densitometer (HOLOGIC, Marlborough, MA, USA), specifically targeting the femoral neck (Figure 1).

### 2.3. Post-Processing

**Figure 2:**
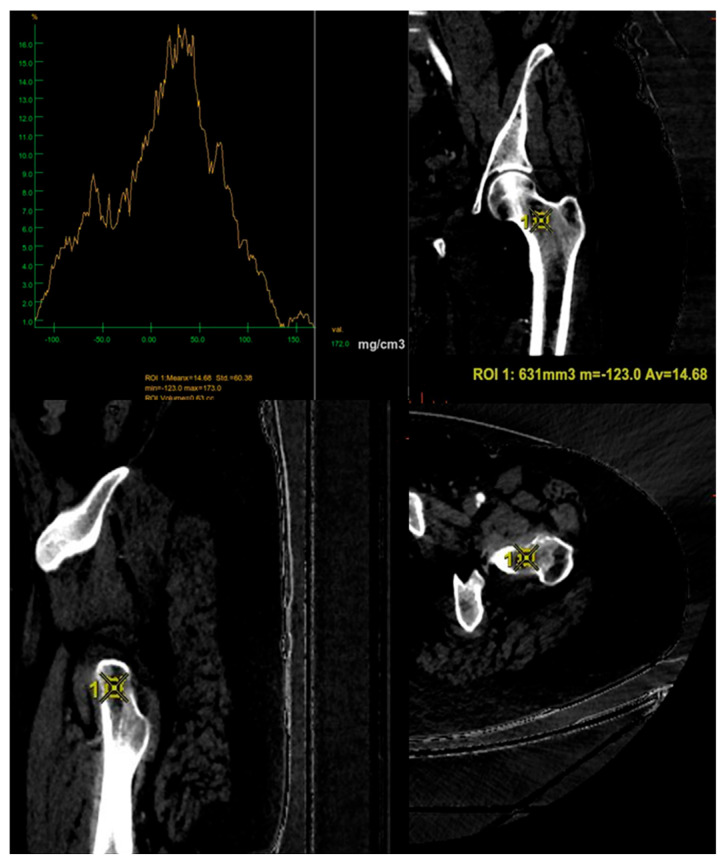
Positioning of the volume of interest (VOI) of 631 mm^3^ in the three spatial planes for the CaOxMono–water base pairing, ensuring exclusion of the cortical bone and inclusion of the trabecular component only. In this case the average value of BMD obtained with these BPMs is 14.68 mg/cm^3^. ROI = region of interest; m = minimum value; Av = average value.

**Figure 3:**
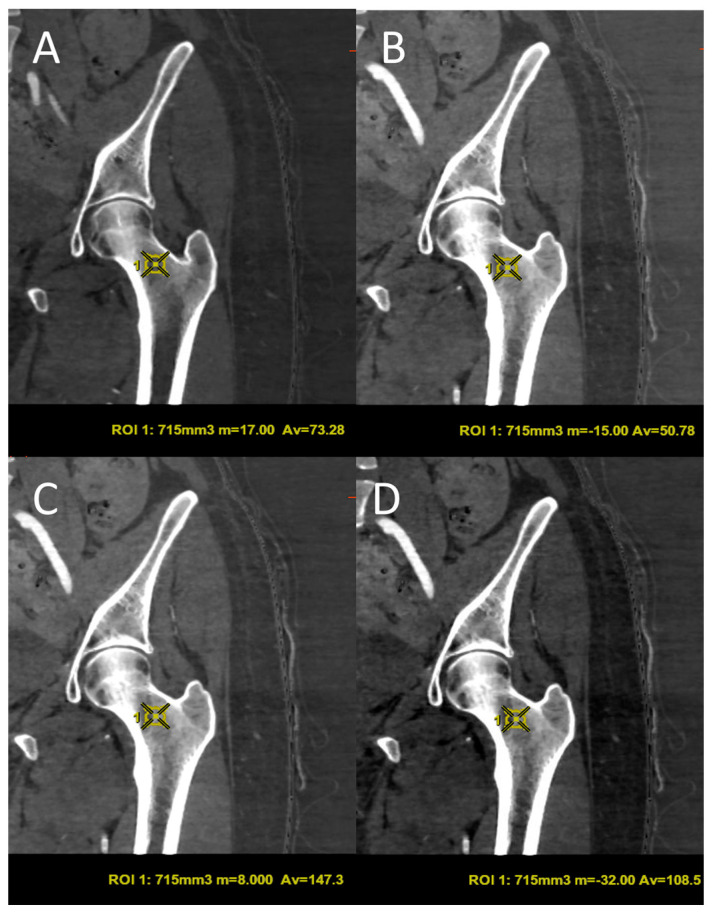
Volume of interest (VOI) of 715 mm^3^ in the coronal plane for different BMPs, such as calcium–fat (**A**), calcium–water (**B**), hydroxyapatite–water (**C**) and hydroxyapatite–fat (**D**). ROI = region of interest; m = medium value; Av = average value.

The GSI images obtained were subsequently processed using AW3.2 software (GE Healthcare, Chicago, IL, USA) with a different combination of BPMs that highlight Carboxymonohydrated (CaOxMono), calcium and hydroxyapatite (HAP) materials. Specifically, carboxymonohydrated density values were measured in CaOxMono–water material decomposition images, hydroxyapatite (HAP) density values were measured in HAP–water and HAP–fat material decomposition images, and calcium density values were measured in calcium–water and calcium–fat material decomposition images. For each combination of BMPs, a three-dimensional volume of interest (VOI) of 715 ± 84 mm^3^ was placed in a uniform and normal-density area of trabecular bone at the femoral neck, excluding the cortical bone component. The VOI comprising the highest trabecular bone content was measured for each patient with the unit mg/cm^3^, representing femoral BMD (Figure 2 and Figure 3). Post-processing measurements were performed by two radiologists with 15 (L.P.S) and 4 (S.P.) years of experience in musculoskeletal imaging, respectively.

### 2.4. Data Evaluation and Statistical Analysis

Statistical analysis was performed to assess the correlation, agreement, and reliability of BMD measurements obtained through BMPs, comparing them to standard DXA.

Statistical analysis was performed using Jamovi software (The jamovi project (2025). jamovi (Version 2.6) [Computer Software]. Retrieved from https://www.jamovi.org (accessed on 30 May 2025)).

Continuous data with a normal distribution are presented as means and standard deviations (mean ± SD). The reproducibility of the measurement was evaluated by the intraclass correlation index (ICC). Spearman’s rank correlation coefficient was used to evaluate the association between BMD values derived from BMPs, including CaOxMono–water, calcium–fat, calcium–water, HAP–fat, and HAP–water. For all correlations, a *p*-value < 0.001 was considered statistically significant. Linear regression plots were used for visualization of the agreement and to identify systematic biases between DECT-derived BMD and DXA values. Statistical significance was set at *p* < 0.05.

## 3. Results

The 41 patients included in this study were divided into risk classes based on DXA values: osteoporosis, osteopenia and normal groups. According to the BMD measurement on DXA, 10 participants were diagnosed as being in the osteoporotic range in the hip area, 15 participants were identified as being in the osteopenic range and 16 subjects were classified as being in the normal range. The mean age of participants in the osteoporotic group was 66.5 ± 11.5 years (58–78), the osteopenic group had a mean age of 59 ± 15 years (44–74), and the normal group had a mean age of 60 ± 16 years (44–76). The osteoporotic group had a mean T-score of −3.1 ± 0.6, the osteopenia group had a mean T-score of −1.65 ± 0.75, and the normal group had a mean T-score of 0.15 ± 0.85. The mean T-scores differed significantly (*p* < 0.001) among the groups.

Similarly, the osteoporotic group had a mean BMD (0.586 ± 0.065 g/cm^2^), the osteopenic group had a mean BMD (0.698 ± 0.08 g/cm^2^), and the normal group had a mean BMD (0.833 ± 0.07 g/cm^2^). The mean BMD differed significantly (*p* < 0.001) among the groups.

The baseline characteristics of the patients included in the study are presented in Table 2.

The reproducibility of the BPMs measurements assessed with ICC, showed all ICC values greater than 0.97. Particularly, the HAP–fat paired base showed the highest ICC value (0.997), while the other bases CaOxMono–water (0.984), calcium–fat (0.983), calcium–water (0.975) and HAP–water (0.983) showed ICCs of 0.975 or higher. The measurements are resumed in Table 3.

Spearman’s correlation demonstrated a strong and statistically significant correlation among BMD and DECT paired bases (*p* < 0.001). The strongest correlations were observed between BMD and calcium–fat (ρ = 0.797). CaOxMono–water (ρ = 0.783), HAP–water (ρ = 0.725), calcium–water (ρ = 0.702) and HAP–fat (ρ = 0.616) also showed strong correlations (Table 4).

**Figure 4:**
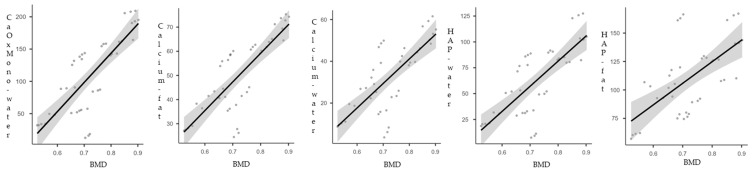
Linear regression plots BMP measurement and BMD. CaOxMono–water = carboxymonohydreated-water; HAP–fat = hydroxyapatite–fat; HAP–water = hydroxyapatite–water.

The relationships between DECT BMPs and BMD measurements were visualized with fitted regression line plots and 95% confidence intervals, consistently demonstrating a linear increase in DECT BMP measurements with increasing BMD values. This observed positive linear trend, along with the narrow confidence intervals, underscores the robustness and consistency of the correlations identified between BMP and BMD levels (Figure 4).

## 4. Discussion

Based on the hypothesis that DXA is subject to several limitations in the assessment of BMD associated with degenerative alterations [18], several studies have demonstrated that DECT is an effective imaging method in the study of BMD [19,20,21].

Furthermore, several comparative studies between CT and DECT have highlighted how DECT shows greater accuracy in BMD assessment compared to conventional single-energy CT [22,23,24,25,26].

Unlike conventional single-energy CT (SECT), DECT operates using two different energy levels. In SECT, the attenuation is measured as the combined result of the photoelectric effect and Compton scatter. So, the measurement can be significantly influenced by technical factors (such as tube voltage settings and imaging protocols) that can affect bone mineral density (BMD) assessments [27].

DECT is capable of independently evaluating the photoelectric and Compton interactions. This separation reduces the influence of technical variables on BMD measurements. Considering that different anatomical structures have different X-ray absorption properties, DECT allows different materials to be distinguished, differentiating bone from other tissues. These characteristics allow an accurate and quantitative analysis of the different tissues [28,29].

Bone is mainly composed of large amounts of bone minerals such as calcium (Ca) and hydroxyapatite (HAP), within which there is red bone marrow, yellow bone marrow (mainly fat), collagen and water. Booz et al. demonstrates that phantomless BMD assessment with DECT of the lumbar spine is significantly more accurate and diagnostic accuracy of osteoporosis compared with Hounsfield Unit (HU) measurements [30]. In fact SETC, in the single-voxel measurement of HU, is influenced by the density of the adipose marrow, since each single voxel captures a mixture of bone and marrow fat [19]. Therefore, hydroxyapatite, a compound formed by calcium and phosphate, seems to better reflect the information on BMD, but it has not yet been demonstrated whether the relative calcium (Ca) content obtained by applying material separation techniques is able to effectively reflect the information on bone density [25].

Wang et al. [12] demonstrated that among the different BPMs for the study of vertebral BMD, the one that has a greater capacity to predict BMD alterations is HAP–water. Several studies have shown that DECT BMD measurement can be highly accurate when using hydroxyapatite and water as reference materials [31,32]. In addition, the use of calcium–water pairs can be useful in detecting age-related changes in the lumbar spine of adult women, showing a significant correlation with BMD values [31,32].

Since hip fractures are one of the most frequent types of fractures in osteoporotic patients, linked to high rates of deformity and disability [33,34], it is appropriate to also evaluate femoral BMD with this method.

It has recently been demonstrated that the use of HAP–fat as BPMs has a high specificity and sensitivity in the assessment of BMD and in the evaluation of fracture risk in postmenopausal women [35].

However, the literature is lacking in studies that have evaluated the use of DECT at the femoral neck. In particular, none of these studies assessed which of the different BMPs had a correlation with DXA values.

In our retrospective study, we demonstrated that it is possible to perform an opportunistic and reliable measurement of femoral BMD in patients undergoing DECT for oncological follow-up. Specifically, the measurements obtained at the femoral neck by means of a three-dimensional VOI using different BPMs are strongly correlated with the BMD values calculated by DXA. In particular, of the different BPMs examined, the base pair that presented a better correlation is calcium–fat (ρ = 0.797).

In contrast to Wang et al.’s demonstration in the vertebral region, where among the different BMPs, the one showing the highest correlation with BMD values was HAP–fat [12], our study revealed that in the femoral region, the BMP showing the highest correlation with the gold standard values was calcium–fat. Although in our previous study, the BMP HAP–fat showed a high specificity and sensitivity in the detection of BMD changes in the femoral neck [35], the results of the present work confirm the correlation between these BPM and BMD values. However, the calcium–fat BMP was found to show an even more significant statistical correlation, suggesting its potential superiority in the assessment of BMD alterations. Therefore, it can be stated that if the BMP HAP–fat is useful in the assessment of vertebral BMD, further studies on the assessment of BMD at the femoral neck level should focus on the usefulness of the BMP calcium–fat.

This result confirms that DECT, through further studies with larger cohorts of patients, can be an effective tool for the diagnosis of BMD alterations. In particular, DECT could be used to simultaneously perform follow-up in chronic patients and calculate BMD, avoiding to perform a DEXA and its consequent exposure to further radiation in order to reduce radiation exposure of these patients [36,37].

As performed in other studies, to avoid possible artifacts related to degenerative alterations or vascular calcifications, we performed measurements only in the trabecular portion [19,38]. In fact, in this study, we demonstrated that by using a three-dimensional VOI that evaluates only the trabecular portion of the femoral neck by standardizing its positioning for the acquisition of measurements, the intra-observer reproducibility of the measurements is very high.

This study has several limitations. First, the small sample size may limit the statistical power and generalizability of our results to a larger population. Furthermore, the small sample limits the validity of our subgroup analyses of patients with osteoporosis, osteopenia, and normal bone density, making our assessments preliminary. Further studies with larger and more diverse cohorts should be performed to confirm these results and improve their applicability. Second, the absence of reference values in the literature regarding BMD calculated with DECT. Third, although other studies suggest that BMD significantly affects bone marrow iodine uptake, supporting the idea that age-related decline in BMD leads to decreased contrast uptake, it should be noted that iodinated contrast agents may still affect the results [39,40]. Since DECT was performed for oncological follow-up after administration of an iodinated contrast medium, this could have influenced BMD estimates, acting as a potential confounder for material decomposition algorithms. Fourth, it should be noted that the study did not assess the risk factors associated with changes in BMD, as patients were included opportunistically during routine diagnostic investigations carried out for other purposes. Therefore, external factors such as medication, comorbidity or other clinical factors were not considered. Fifth, reproduction of our results is hampered by the fact that some BPMs are vendor-specific and only usable on certain DECTs, limiting the ability to validate the results with other vendors’ DECTs.

## 5. Conclusions

As in other previous studies, we confirmed the potential opportunistic use of DECT for BMD measurement in patients undergoing oncological follow-up. In particular, through the use of different BPMs, there is a strong and significant statistical correlation with the gold standard values (DXA) and a high intra-observer reproducibility of the data obtained. Among the different BPMs, those that have a higher statistical correlation are calcium–fat (ρ = 0.797) and CaOxMono–water (ρ = 0.783). Furthermore, these have shown a strong intra-observer reproducibility with ICC values of 0.983 and 0.984, respectively. However, our study has several limitations, including the small sample size and the lack of standard references of BPMs values for the stratification of patients into the three risk classes (osteoporosis, osteopenia and normal). Therefore, further studies defining standard reference values for the various BPMs with larger samples are needed to confirm our results.

## Figures and Tables

**Table 1:** Criteria for inclusion and exclusion.

Inclusion Criteria	Exclusion Criteria
Postmenopausal females	Previous fractures or bone lesions
Patients in oncological follow-up	Presence of metal prosthesis
Patients with maximum gap of 6 months between DXA and DECT	Post-surgical patients

**Table 2:** Characterization of the study population. CaOxMono–water = carboxymonohydreated–water; HAP–fat = hydroxyapatite–fat; HAP–water = hydroxyapatite–water.

Characteristics	Osteoporosis	Osteopenia	Normal
Mean age (range)	66.5 ± 11.5 (58–78)	59 ± 15 (44–74)	60 ± 16 (44–76)
Patients	10	15	16
T-score	−3.1 ± 0.6	−1.65 ± 0.75	0.15 ± 0.85
BMD (g/cm^2^)	0.586 ± 0.065	0.698 ± 0.08	0.833 ± 0.07
CaOxMono–water (g/cm^3^)	50.93 ± 22.8	83.92 ± 47.5	167.07 ± 32.2
Calcium–Fat (g/cm^3^)	34.68 ± 6.6	43.84 ± 12.1	65.1 ± 9.2
Calcium–water (g/cm^3^)	17.12 ± 9.2	26.13 ± 15.0	46.7 ± 7.4
HAP–Fat (g/cm^3^)	131.4 ± 21.2	105.19 ± 32.6	82.06 ± 21.3
HAP–water (g/cm^3^)	30.58 ± 12.7	49.81 ± 28.7	94.1 ± 19.7

**Table 3:** Intraclass correlation coefficients (ICCs) for the assessment of measurement’s reproducibility. CaOxMono–water = carboxymonohydreated–water; HAP–fat = hydroxyapatite–fat; HAP–water = hydroxyapatite–water.

	CaOxMono–Water	Calcium–Fat	Calcium–Water	HAP–Fat	HAP–Water
ICC	0.984	0.983	0.975	0.997	0.983

**Table 4:** Spearman’s correlation coefficients between DXA values of BMD and BMPs values. CaOxMono–water = carboxymonohydreated–water; HAP–fat = hydroxyapatite–fat; HAP–water = hydroxyapatite–water.

		CaOxMono–Water	Calcium–Fat	Calcium–Water	HAP–Fat	HAP–Water
BMD	Spearman’s rho	0.783	0.797	0.702	0.616	0.725
	*p*-value	<0.001	<0.001	<0.001	<0.001	<0.001

## Data Availability

The data presented in this study are available upon request from the corresponding author.
